# Dual actions of gallic acid and andrographolide trigger AdipoR1 to stimulate insulin secretion in a streptozotocin-induced diabetes rat model

**DOI:** 10.1016/j.jtcme.2022.09.002

**Published:** 2022-09-28

**Authors:** Tet Soon Wong, Fatahiya Mohamed Tap, Zanariah Hashim, Fadzilah Adibah Abdul Majid, Nor Hafizah Zakaria, Parsaoran Siahaan, Abeer Mogadem

**Affiliations:** a School of Chemical and Energy Engineering, Universiti Teknologi Malaysia, 81310, Johor, Malaysia; b School of Chemical Engineering, College of Engineering, Universiti Teknologi MARA Bukit Besi, 23200, Dungun, Terengganu, Malaysia; c Institute of Marine Biotechnology, Universiti Malaysia Terengganu, 21030, Kuala Nerus, Terengganu, Malaysia; d Department of Chemistry, Diponegoro University, Semarang, Indonesia; e Chemistry Department, Taibah University, Al-Madinah Al-Munawarah, Saudi Arabia

**Keywords:** Gallic acid, Andrographolide, adipoR1, Insulin, Diabetes mellitus, STZ, Streptozotocin, GLUT4, Glucose transporter-4, AdipoR1, Adiponectin receptor 1, GA, Gallic acid, AGP, Andrographolide, PBS, Phosphate buffer saline, H&E, Hematoxylin-eosin, ELISA, Enzyme-linked immunosorbent assay, TBST, Tris-buffered saline with 0.1% (v/v) Tween-20

## Abstract

Common treatments for the management of diabetes have limitations due to side effects, hence the need for continuous research to discover new remedies with better therapeutic efficacy. Previously, we have reported that the combination treatment of gallic acid (20 mg/kg) and andrographolide (10 mg/kg) for 15 days demonstrated synergistic hypoglycemic activity in the streptozotocin (STZ)-induced insulin-deficient diabetes rat model. Here, we attempt to further elucidate the effect of this combination therapy at the biochemical, histological and molecular levels. Our biochemical analyses showed that the combination treatment significantly increased the serum insulin level and decreased the total cholesterol and triglyceride level of the diabetic animals. Histological examinations of H&E stained pancreas, liver, kidney and adipose tissues of combination-treated diabetic animals showed restoration to the normalcy of the tissues. Besides, the combination treatment significantly enhanced the level of glucose transporter-4 (GLUT4) protein expression in the skeletal muscle of treated diabetic animals compared to single compound treated and untreated diabetic animals. The molecular docking analysis on the interaction of gallic acid and/or andrographolide with the adiponectin receptor 1 (AdipoR1), a key component in the regulation of pancreatic insulin secretion, revealed a greater binding affinity of AdipoR1 to both compounds compared to individual compounds. Taken together, these findings suggest the combination of gallic acid and andrographolide as a potent therapy for the management of diabetes mellitus.

## Highlights

• The combined GA and AGP treatment in diabetic rats rescued the pancreas, liver and kidney from tissue degradation.
• The combined treatment in diabetic rats reduced total cholesterol and triglyceride levels.
• Administration of both GA and AGP to diabetic rats significantly restored GLUT4 protein expression to its near-normal level.
• Molecular docking analysis showed favorable binding affinity of both compounds on adiponectin receptor AdipoR1.

## Graphical abstract

**Figure:**
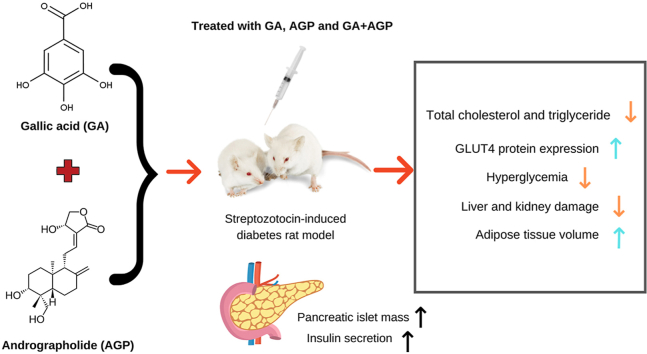

## 1 Introduction

Diabetes mellitus, commonly known as diabetes, is a metabolic disorder characterized by high blood sugar levels (hyperglycemia) due to defects in insulin secretion, insulin action, or both.^1^ According to the International Diabetes Federation, about 537 million adults aged 20–79 years around the world have diabetes in 2021, and the number is expected to increase to 783 million in 2045 without effective interventions.^2^ Current oral antidiabetic drugs for the management of diabetes have limitations due to side effects such as weight gain,^3^ severe hypoglycemia,^4^ secondary failure,^5^^,^^6^ and gastrointestinal disorders, such as nausea, vomiting and diarrhea.^7^ Hence, there is a need to discover new antidiabetic agents with better therapeutic efficacy and fewer side effects.

Levels of adiponectin were found to be low in diabetic patients,^8^ as well as in streptozotocin (STZ)-induced diabetic rats.^9^ *In vitro* and animal studies have shown that the administration of adiponectin increased β-cell viability and insulin secretion.^10^^,^^11^ However, due to difficulties in the production of biologically active adiponectin, treatment with adiponectin receptor agonists, which mimic adiponectin action in activating the downstream signaling pathway of adiponectin, can be an alternative and viable therapeutic approach in the treatment of diabetes.

Phytochemicals that are abundant in plants have been consumed for centuries to treat hyperglycemic conditions.^12^ Interest in the traditional use of natural products to treat diabetes has led researchers to scientifically validate their efficacy and safety profile.^13^, ^14^, ^15^ Earlier studies demonstrated that gallic acid (GA), a bioactive polyphenol, and andrographolide (AGP), a major bioactive compound of *Andrographis paniculata*, lessened hyperglycemia in diabetic rats and reversed pancreatic β-cells damage.^16^^,^^17^ We have previously reported that the combination therapy at a 2:1 GA:AGP combination ratio demonstrated synergistic hypoglycemic activity in the STZ-induced insulin-deficient diabetic rats.^18^ The present study aimed to investigate the effect of this combination therapy on the serum insulin, total cholesterol, and triglyceride level, as well as on the histology of the pancreatic, liver, kidney and adipose tissues of the STZ-induced diabetic rats. In addition, the effect of the treatment on the protein expression level of glucose transporter-4 (GLUT4) in the skeletal muscle was assessed. We also evaluated the association of the combination treatment compounds with adiponectin receptor 1 (AdipoR1) using *In silico* molecular docking studies.

## 2 Materials and methods

### 2.1 Materials

Streptozotocin (STZ), gallic acid, andrographolide, neutral buffered formalin, Harris hematoxylin solution, eosin Y, and alkaline phosphatase-conjugated goat anti-rabbit secondary antibody were purchased from Sigma-Aldrich (Selangor, Malaysia). Sodium azide and all organic solvents were purchased from Merck (Selangor, Malaysia). Rat Insulin ELISA kit was purchased from Mercodia (Sweden). CHOL2 total cholesterol test kit and TRIGL (GPO-PAP) triglyceride test kit were procured from Roche (Germany). RIPA lysis buffer, 2 × protein loading buffer, Tris-buffered saline with 0.1% (v/v) Tween-20, and BCIP/NBT chromogenic substrate kit were purchased from Solarbio (China). PVDF membrane was procured from Bio-Rad (USA). Rabbit anti-rat GLUT4 primary antibody was procured from Cusabio (China).

### 2.2 Collection of blood and tissue samples

Blood and tissue samples from the animal experiments reported in our previous study^18^ following 15 days of treatment with vehicle, gallic acid (GA) and/or andrographolide (AGP) on the normal control and STZ-induced diabetic rats (40 mg/kg BW STZ, dissolved in 0.1 M cold citrate buffer, pH 4.5) were used for further analyses in this present study. The samples were taken from five groups of rats (n = 5–6):

Group 1: Normal control rats received vehicle alone (10 mL/kg distilled water).

Group 2: Diabetic control rats received vehicle alone (10 mL/kg distilled water).

Group 3: Diabetic rats given commercially obtained GA at 20 mg/kg BW.

Group 4: Diabetic rats given commercially obtained AGP at 10 mg/kg BW.

Group 5: Diabetic rats given combination of GA + AGP (2:1) at 20 mg/kg:10 mg/kg.

Blood samples taken from the cardiac puncture during the animal termination procedure were left to clot for 30 min at room temperature and then centrifuged at 1500×*g* for 10 min at 4 °C. Following centrifugation, serum was aliquoted into several clean tubes and kept at −80 °C for biochemical analysis.

Upon termination of the experimental rats, the pancreas, liver, kidney, adipose tissues and soleus muscles were immediately removed and washed briefly in chilled saline. The tissues were cut into smaller sizes, with some portions immediately snap-frozen in liquid nitrogen and stored at −80 °C for other assays, while the other portions were fixed in 10% neutral buffered formalin at the volume of at least 10 × that of the tissues and kept overnight with gentle agitation at 4 °C, followed by washing in cold phosphate buffer saline (PBS) (20 min, 3 times) and stored in PBS with sodium azide (0.01% w/v) at 4 °C until analyses. This study has received ethical approval from the Universiti Kebangsaan Malaysia Animal Ethical Committee (Approval Code: UTM/2016/FADZILAH/28-JAN./723-APR.-2016-APR.-2019).

### 2.3 Biochemical analysis

Serum insulin level was assayed by a rat insulin assay kit via enzyme-linked immunosorbent assay (ELISA) and the absorbance was measured using the ELx800 Absorbance Microplate Reader (BioTek Instruments, USA). Serum levels of total cholesterol and triglyceride were assayed by a total cholesterol test kit and a triglyceride test kit, respectively and analyzed with the Cobas c 111 clinical chemistry analyzer (Roche, Germany) according to the manufacturer's instructions.

### 2.4 Histopathological studies

The formalin-fixed pancreatic, liver, kidney and adipose tissues were dehydrated in a graded series of ethanol and were embedded in paraffin wax. Tissue sections (6 μm thick) were attained using a microtome (Leica) and stained with hematoxylin-eosin (H&E). The tissue sections were viewed under a light microscope (Olympus).

### 2.5 Western blot analysis

Total protein from the soleus muscle was obtained by grinding the frozen tissue into a fine powder with liquid nitrogen using a mortar and pestle, and then incubating the powdered tissue in RIPA lysis buffer (containing PMSF protease inhibitor) on ice for 30 min. After centrifugation of the homogenate at 14000×*g* for 5 min at 4 °C, the supernatant was mixed with an equal amount of 2 × protein loading buffer (containing β-mercaptoethanol). Total protein (80 μg) was separated on 10% SDS-polyacrylamide gel and transferred onto PVDF membrane, using standard protocols. Membranes were stained with Ponceau S (0.5% w/v, 1% v/v acetic acid) by immersing the membrane in the stain for 5 min, followed by rinsing with distilled water to remove the background stain. After visualizing and capturing the total protein load, the stain was removed by immersing the membrane in 0.1 M NaOH until the red dye was removed, followed by rinsing the membrane in distilled water four times. Membranes were then blocked with 3% bovine serum albumin in Tris-buffered saline with 0.1% (v/v) Tween-20 (TBST) and incubated with a primary antibody detecting GLUT4 at a 1:500 dilution. After washing three times each for 5 min in TBST, the membranes were incubated with alkaline phosphatase-conjugated goat anti-rabbit secondary antibody at a 1:10000 dilution for 1 h. The blots so obtained were washed with TBST three more times, each 5 min, and the expressions were detected by using BCIP/NBT chromogenic substrate kit. As a loading control, the band densitometry was normalized to the total protein stained with Ponceau S before the antibody incubation. Densitometry analysis was performed using Image Studio Lite software.

### 2.6 Molecular docking studies

#### 2.6.1 Single docking

The three-dimensional crystal structure of adiponectin receptor 1, AdipoR1 (PDB ID: 5LXG) was obtained from the Protein Data Bank (PDB) (https://www.rcsb.org/) as a target receptor for the docking studies. The grid dimension of 5LXG was set at 24 x 28 x 28 according to coordinates x, y, and z for the target binding sites. The central cavity within the transmembrane opening of AdipoR1 was selected as the docking region because this region was reported to have high druggability values.^19^ Hydrogen bonds and computed Gasteiger charges were added to the protein by using Autodock Tools 1.5.7. The ligand structures of andrographolide and gallic acid were obtained from PubMed Database (https://www.ncbi.nlm.nih.gov/pubmed/). The ligands minimization and preparation were performed using LigPrep and Autodock Tools 1.5.7, respectively.^20^ LigPrep determined all tautomeric states, one stereoisomeric state, and one low energy ring conformation for each ligand. The OPLS_2005 force field was used to optimize the structure. Molecular docking simulation was performed using Autodock Vina^21^ to find the ligand pose with the lowest predicted free energy of binding. The results were analyzed using Discovery Studio Visualizer, PyMOL and UCSF Chimera to view and represent the molecular interactions.

#### 2.6.2 Sequential docking

In this study, two types of sequential docking were investigated. Firstly, GA was docked with AdipoR1. The complex of GA with AdipoR1 was saved as a single file, where the compound of GA was considered as part of the receptor. Docking was then carried out on this complex with AGP as the second compound. All the parameters used in this docking approach were similar to the parameter in the single docking method. Secondly, AGP was docked with AdipoR1 and the complex was saved as a single file. Docking was then carried out on this complex with GA as the second compound.

## 3 Results

### 3.1 Effect of treatment on insulin, total cholesterol and triglyceride

**Fig. 1:**
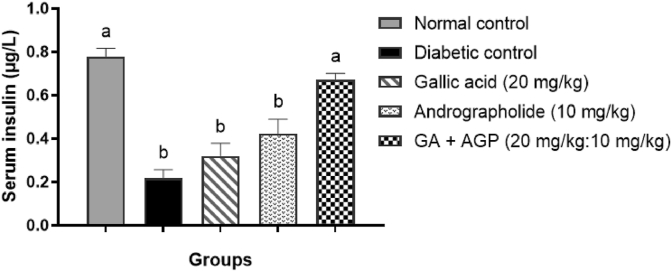
Effect on serum insulin levels after respective treatment for 15 days. Bars are represented as mean ± S.E.M for diabetic control group (n = 5) and other groups (n = 6). Different letters indicate significant differences analyzed by one-way ANOVA followed by the Tukey post hoc test (*P* < 0.05).

In the diabetic control group, insulin levels in serum were reduced significantly (*P* < 0.05) compared with the normal control group (Fig. 1). However, after administration of GA + AGP (20 mg/kg:10 mg/kg) to diabetic rats, insulin levels were improved significantly (*P* < 0.05) compared with the diabetic control group.

In the diabetic control group, total cholesterol and triglyceride levels in serum were increased significantly (*P* < 0.05) compared with the normal control group. However, after administration of GA + AGP (20 mg/kg:10 mg/kg) to diabetic rats, total cholesterol and triglyceride levels were significantly (*P* < 0.05) decreased compared with diabetic control rats (Table 1).

**Table 1:** Total cholesterol and triglyceride level in experimental rats after 15 days of treatment with GA and AGP.

Groups	n	Total Cholesterol (mM)	Triglyceride (mM)
Normal control	6	1.30 ± 0.04^b^	0.65 ± 0.02^b^
Diabetic control	5	1.86 ± 0.02^a^	1.46 ± 0.02^a^
GA (20 mg/kg)	6	1.57 ± 0.03^b^	0.80 ± 0.06^b^
AGP (10 mg/kg)	6	1.50 ± 0.15^b^	0.73 ± 0.08^b^
GA + AGP (20 mg/kg:10 mg/kg)	6	1.50 ± 0.04^b^	0.70 ± 0.04^b^

Values indicate mean ± S.E.M. Superscript a means *P* < 0.05, compared with normal control values. Superscript b means *P* < 0.05, compared with diabetic control values.

### 3.2 Histopathological examination

**Fig. 2:**
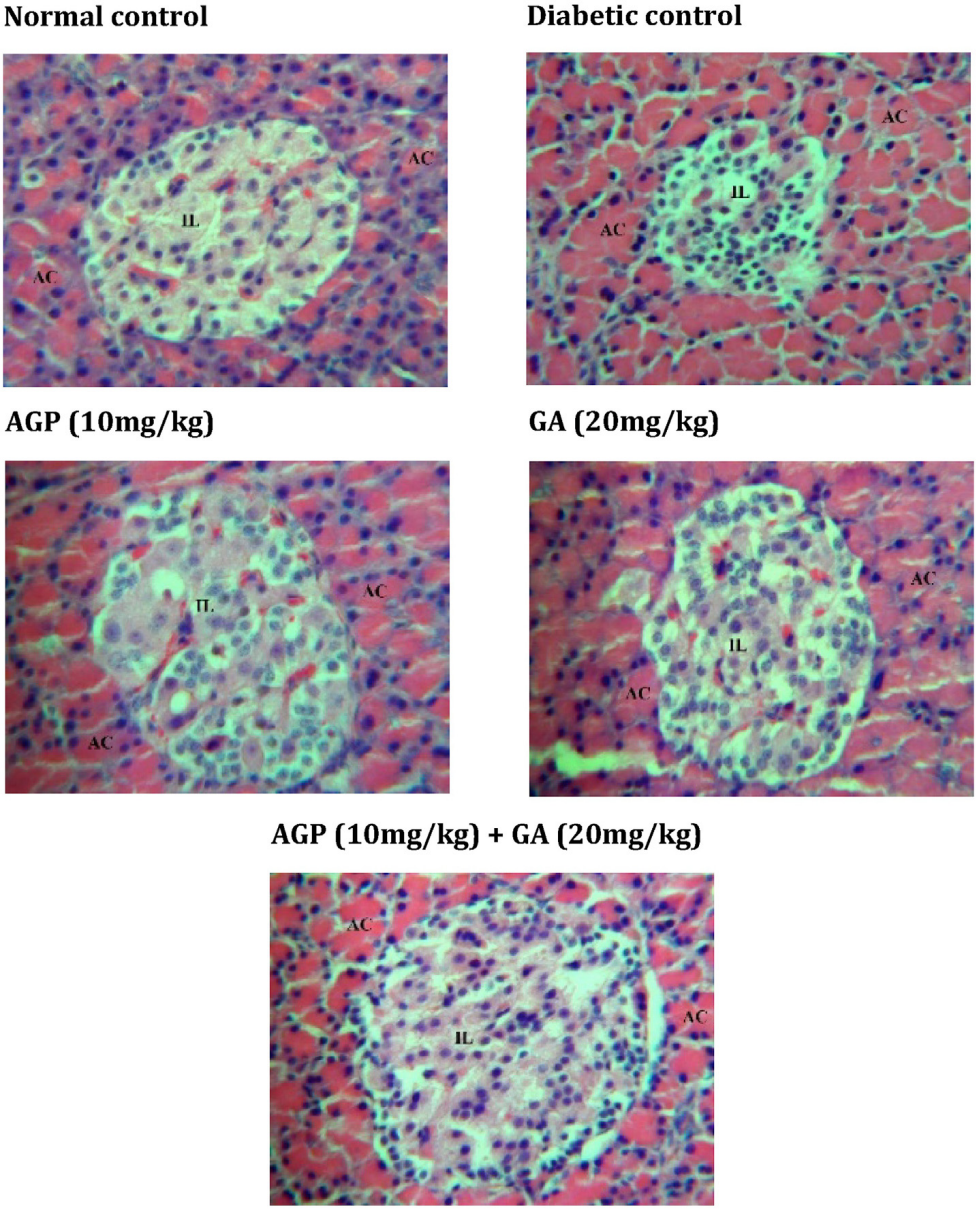
Light photomicrograph of the pancreatic section from experimental rats stained with H&E. **AC** = acinar cells; **IL** = islets of Langerhans. The image is representative of three animals per experimental group (magnification × 200).

**Fig. 3:**
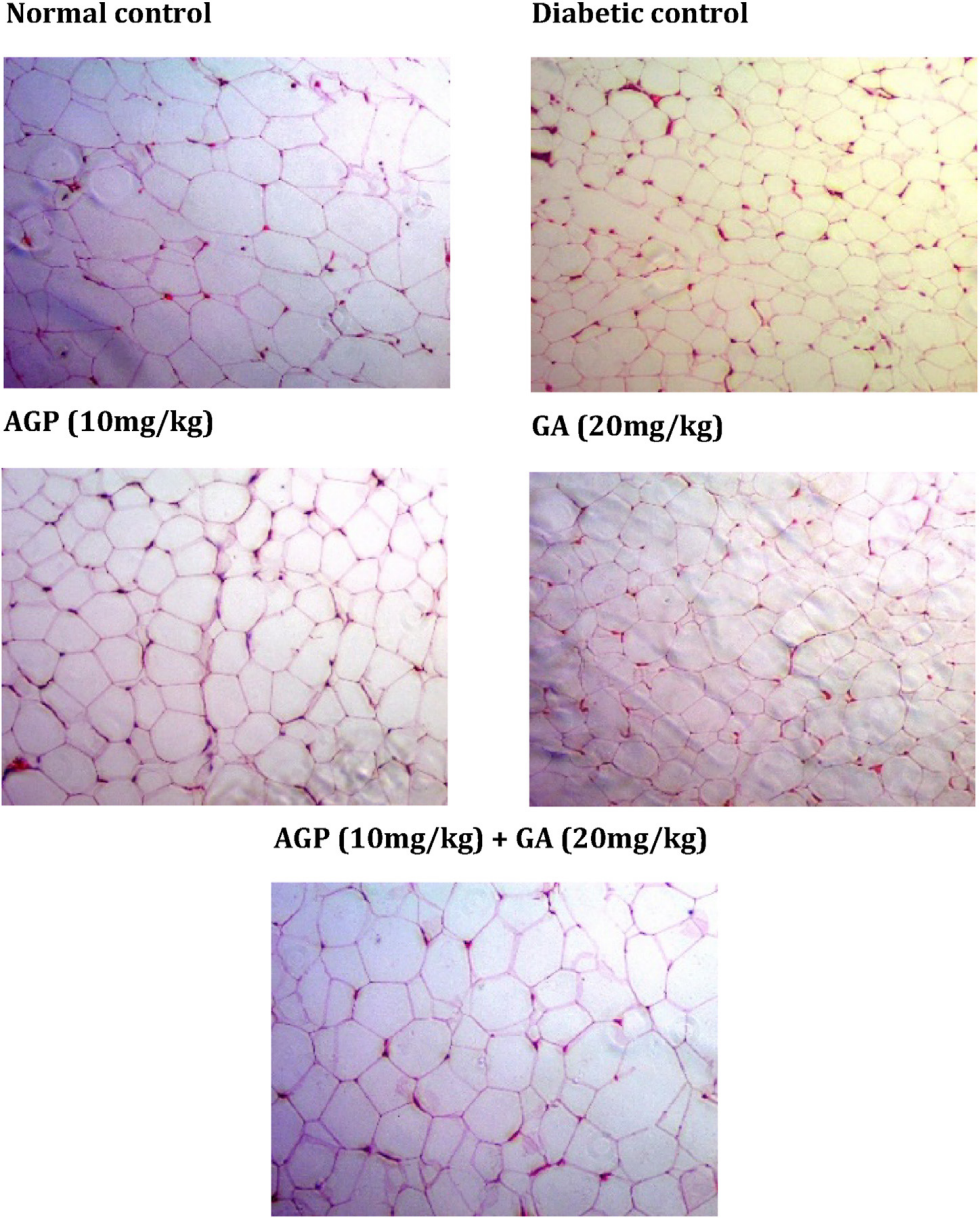
Light photomicrograph of H&E-stained adipose tissue from experimental rats. The image is representative of three animals per experimental group (magnification × 100).

**Fig. 4:**
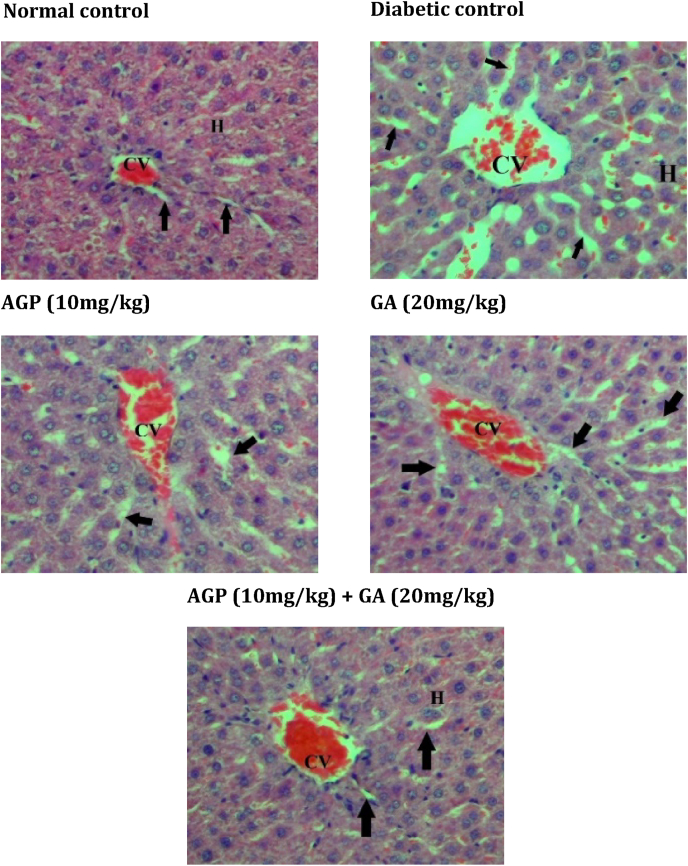
Light photomicrograph of H&E-stained liver section from experimental rats. **CV** = central vein; **H** = hepatocytes; **black arrow** = sinusoids. The image is representative of three animals per experimental group (magnification × 200).

**Fig. 5:**
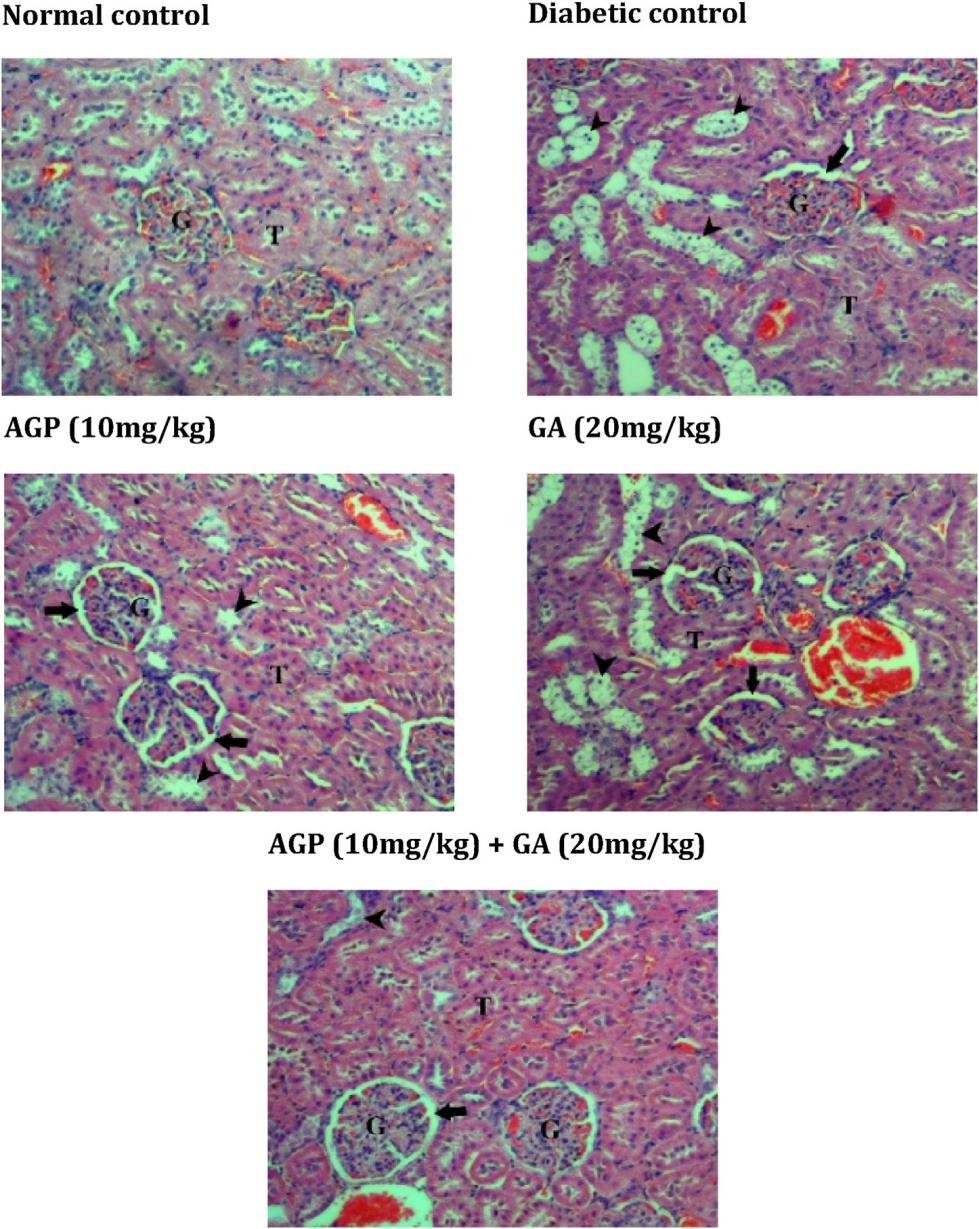
Light photomicrograph of H&E-stained kidney section from experimental rats. **T** = renal tubules; **G** = glomerulus; **black arrow** = Bowman's space; **arrowhead** = vacuolar degeneration of the renal tubules. The image is representative of three animals per experimental group (magnification × 100).

Histological examinations of the pancreatic islet, liver, kidney and adipose tissues of experimental rats stained with hematoxylin and eosin (H&E) are presented in Fig. 2, Fig. 3, Fig. 4, Fig. 5. Diabetic control rats exhibited severe islet shrinkage and structure distortion, with degenerative and necrotic changes of the islet cells, along with enlargement of the surrounding acinar cells.

The liver tissues of diabetic control rats showed dilatation of the sinusoids near the central vein. Diabetic control rats also exhibited vacuolar degeneration of the renal tubules and an increase of the Bowman's space in the kidney. In addition, the adipocytes of the diabetic control group were relatively smaller in size compared to the normal control group.

In contrast, AGP (10 mg/kg) treated and GA (20 mg/kg) treated diabetic rats showed slight recovery of pancreatic islets, liver, kidney and adipose tissue architectures. In striking contrast, GA + AGP (20 mg/kg:10 mg/kg) treated diabetic rats showed the near-normal histological appearance of pancreatic islets, liver, kidney, and adipose tissue.

### 3.3 Effect of treatment on GLUT4 protein expression in skeletal muscle

**Fig. 6:**
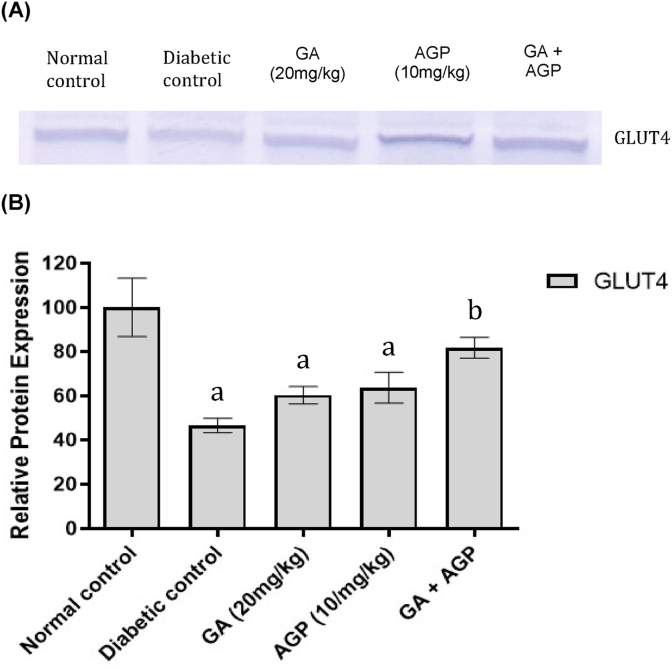
Effects of gallic acid and andrographolide treatment on GLUT4 protein expression in soleus muscle. (A) shows representative GLUT4 protein bands; (B) shows the relative GLUT4 protein expression normalized by total protein. (Lane 1) Normal control, (Lane 2) diabetic control, (Lane 3) diabetic + gallic acid (20 mg/kg), (Lane 4) diabetic + andrographolide (10 mg/kg), and (Lane 5) diabetic + gallic acid (20 mg/kg) + andrographolide (10 mg/kg). Bars are represented as means ± S.E.M for three independent experiments performed. ^a^ significantly different from the normal control group (*P* < 0.05); ^b^ significantly different from the diabetic control group (*P* < 0.05).

GLUT4 protein expression level in the skeletal muscle of the diabetic control group decreased significantly (*P* < 0.05) compared to the normal control group (Fig. 6). Treatment with either GA (20 mg/kg) or AGP (10 mg/kg) did not significantly alter the GLUT4 protein expression level of the diabetic rats. On the other hand, treatment of the diabetic rats with a combination of GA (20 mg/kg) and AGP (10 mg/kg) increased the GLUT4 protein expression level significantly (*P* < 0.05) compared to the vehicle-treated diabetic control group.

### 3.4 Molecular docking analysis

The binding affinity of the compounds ranges from −5.3 kcal/mol to −7.6 kcal/mol (Table 2, Suppl. 1). In the case of a single ligand-receptor complex, the result showed good binding energy for each ligand. However, the combination of GA and AGP ligands towards AdipoR1 has a better binding affinity compared to the single ligand when GA is docked first, followed by AGP. The positions of ligands docked were different according to the method of docking. The single docking approach showed that both ligands were docked at the same binding site (Fig. 7A, Suppl. 2). Gallic acid forms four hydrogen bonds with residues Gln335, Glu134, Asn137, and Arg320 and one hydrophobic bond with residue His337 on AdipoR1 (Fig. 7B, Suppl. 2), whereas andrographolide forms three hydrogen bonds with residues Glu134, Tyr194 and His337 (Fig. 7C, Suppl. 2). On the other hand, the sequential docking showed that the ligands were docked at different sites (Fig. 8, Suppl. 3 and Fig. 9, Suppl. 4). In the sequential docking of gallic acid followed by andrographolide, andrographolide forms one hydrophobic bond with residue Tyr209 and two hydrogen bonds with residues Asp106 and Asp208 on AdipoR1 (Fig. 8B, Suppl. 3), whereas in the sequential docking of andrographolide followed by gallic acid, gallic acid forms two hydrophobic bonds with residues Ile212 and Phe271, and two hydrogen bonds with residue Tyr209 (Fig. 9B, Suppl. 4).

## 4 Discussion

In our animal experiments, we used STZ to induce diabetes because STZ selectively destroys pancreatic islet β cells via the GLUT2 glucose transporter, which resulted in animals experiencing general characteristics of human diabetes such as hyperglycemia, polyuria, polydipsia and hyperphagia.^22^^,^^23^ The STZ-induced diabetic animal model has been very useful in elucidating the mechanisms of diabetic pathogenesis and in screening potential natural products and pharmacological agents for anti-diabetic properties.^22^^,^^24^ This diabetic rodent model is also considered to be more economical and rapid for preliminary testing of antidiabetic agents compared to other models.^25^

Hyperglycemia can lead to a rise in free radicals production and form oxidative stress, which damages tissues such as the pancreas.^26^ This is due to the low levels of antioxidative enzymes in pancreatic β cells which makes them extremely susceptible to oxidative stress damage in the pathology of diabetes.^27^ Treatments with antioxidant supplements have been shown to reduce oxidative stress and improve pancreatic STZ-induced diabetes animal model, STZ partially destructs the pancreas and reduces β cell mass proliferation and function in type 2 diabetes.^28^ The cytotoxicity of STZ towards pancreatic β cells is mediated by reactive oxygen species (ROS).^23^ Earlier studies reported that gallic acid demonstrated antioxidant effects and reversal of pancreatic dysfunction in STZ-induced diabetic rats.^29^^,^^30^ Similarly, andrographolide also possesses antioxidant activity, and several studies had shown that it is capable of lowering blood glucose, leading to a reduction of the damaging ROS and thereby protecting β cells from further damage and facilitating their regeneration in diabetic animals.^17^^,^^31^^,^^32^ In the present study, administration of both gallic acid and andrographolide to the diabetic rats showed significant improvement in serum insulin to near normal level compared to treatment by gallic acid alone, andrographolide alone, or vehicle-treated diabetic control rats. This result suggested improvement in the β cells insulin secretion function, which supported by the recovery of pancreatic islets morphology seen in our histological analysis for the combination treatment of diabetic rats. We theorize that the synergistic blood glucose reduction activity of the combined gallic acid and andrographolide treatment reported in our previous study,^18^ leads to a reduction of oxidative stress and thereby enabled the recovery of pancreatic islets and their subsequent insulin secretion function.

Oxidative stress in diabetes also impairs other vital organs such as liver^33^ and kidney.^34^ Previous studies have shown that treatment with antioxidant agents decreased hyperglycemia and oxidative stress, and ameliorated diabetic complications of the liver^35^ and kidney.^34^ As supported by our histological data, administration of both gallic acid and andrographolide in diabetic rats rescued the liver and kidney from tissue degradation.

Due to a lack of insulin levels or action, diabetes prevents the body from getting sufficient glucose from the blood into the body's cells to be converted into energy. To compensate, the body starts burning fat stores and muscle for energy,^36^ causing a reduction in overall body weight such as that observed in the untreated diabetic rats in the current study. Administration of both gallic acid and andrographolide to diabetic rats reduced severe weight loss compared to untreated diabetic control rats (data not shown). This observation was supported by our histological data demonstrating improvement in the adipose tissue volume of the combination-treated diabetic rats.

In the current study, STZ-induced diabetic rats exhibited lipid abnormalities, characterized by higher levels of total cholesterol and triglyceride, which supported the findings of other investigators that hyperlipidemia is associated with diabetes mellitus.^37^^,^^38^ Previous study by Young et al*.*^39^ reported that hypercholesterolemia is caused by elevated cholesterol absorption in the intestine due to insulin deficiency. Studies also have shown that high cholesterol and triglyceride levels can be reduced by insulin treatment.^40^^,^^41^ Therefore, the improvement of insulin levels from the treatment of both gallic acid and andrographolide in diabetic rats is correlated with the reduction of total cholesterol and triglyceride levels in the current study.

GLUT4 is part of a family of glucose transporter proteins expressed primarily in skeletal muscle and adipose tissue.^42^ It plays a key role in insulin-stimulated glucose uptake into muscle and adipose tissue.^43^ A previous study by Kahn et al.^44^ reported that the GLUT4 protein level in skeletal muscle of streptozotocin-induced diabetic rats reduced significantly after 14 days of hyperglycemia state, which is consistent with the data in the present study. Administration of both gallic acid and andrographolide to diabetic rats significantly restored GLUT4 protein expression to its near normal level, which was correlated with the restoration of serum insulin to near normal control level. Our results are in line with a prior report's findings that insulin therapy restored glucose transport activity and GLUT4 protein level in the soleus muscle of streptozotocin-induced diabetic rats.^45^ Therefore, we postulate that the combined treatment of gallic acid and andrographolide could facilitate the recovery of pancreatic β cells, which leads to improved insulin levels in circulation and subsequently increased GLUT4 protein levels in the soleus muscle.

The cell signaling cytokine produced by adipose tissue, adiponectin, is a key regulator of insulin secretion^10^ and insulin sensitivity.^46^ In pancreatic β cells, adiponectin exerts its biological activity by activating two receptors isoform, adiponectin receptor 1 (AdipoR1), the predominant isoform expressed in β cells,^11^^,^^47^ and adiponectin receptor 2 (AdipoR2).^48^ The administration of adiponectin had been shown to increase β cell viability and insulin secretion *in vitro* and *in vivo*.^10^^,^^11^ In the present study, the better binding affinity of both gallic acid and andrographolide on AdipoR1 compared to individual compounds observed in current molecular docking analysis, as well as the pancreatic islet cell regeneration and insulin augmentation observed in the combined treatment by gallic acid and andrographolide in diabetic rats suggest the potential use of these compounds as AdipoR1 agonists.

However, the effect of this binding activity on the downstream signaling proteins of AdipoR1 is unclear. The difference in the docking site of the ligands for sequential docking shown in the present molecular docking study might be due to the allosteric location of the binding site on AdipoR1, which might contribute to the improved proliferation of the pancreatic β cells and their subsequent insulin secretion. Further *in vitro*, cell-based studies are warranted to validate the benefit of this binding mechanism.

## 5 Conclusions

In conclusion, combination therapy with gallic acid and andrographolide ameliorated diabetes complications via regeneration of pancreatic islets and protection of kidney and liver from tissue degradation. The regeneration of the pancreatic islets leads to the increase in insulin secretion, improvement of lipid profile and upregulation of GLUT4 protein expression in skeletal muscles. The better binding affinity of gallic acid and andrographolide on adiponectin receptor AdipoR1 might be the contributing factor to the amelioration of diabetogenic conditions caused by STZ treatment. Taken together, these results suggest that combination treatment with gallic acid and andrographolide warrants further study as a potential therapy in the management of diabetes mellitus and its secondary complications.

## Novelties

To the best of our knowledge, this is the first report on the effect of combination treatment of GA and AGP on diabetes-related parameters in a diabetic rat model. This report expands our understanding of the synergistic antihyperglycemic activity of the combined treatment of GA and AGP in a diabetic rat model.

## Declaration of competing interest

The authors want to declare that they have no conflict of interest associated with this publication.
