# Supplementary material for: Dual actions of gallic acid and andrographolide trigger AdipoR1 to stimulate insulin secretion in a streptozotocin-induced diabetes rat model
Source: J Tradit Complement Med. 2022 Sep 28;13(1):11–9. doi: 10.1016/j.jtcme.2022.09.002 (PMC9845651; doi:10.1016/j.jtcme.2022.09.002)
Supplement: Multimedia component 1 [file mmc1.doc]

**Supplementary 1:**

**Table 2. The binding affinities for the compounds.**

| Compound name | Binding energy (Kcal/mol) |
| --- | --- |
| Gallic acid (GA) | -6.0 |
| Andrographolide (AGP) | -7.1 |
| GA+AGP | -7.6 |
| AGP+GA | -5.3 |

**Supplementary 2:**


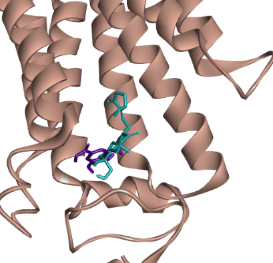

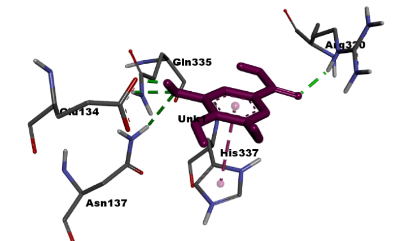

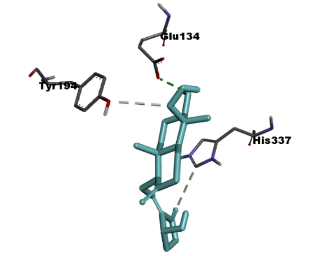


**A)**

**B)**

**C)**

**Fig. 7** The position of gallic acid (purple) and andrographolide (cyan) on AdipoR1 based on the single docking method (A). Molecular interaction showing hydrogen bond (green dash line) and hydrophobic bond (purple dash line) of gallic acid (B) and andrographolide (C) with AdipoR1 residues.

**Supplementary 3:**


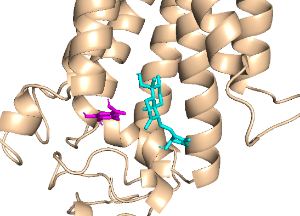


**A)**


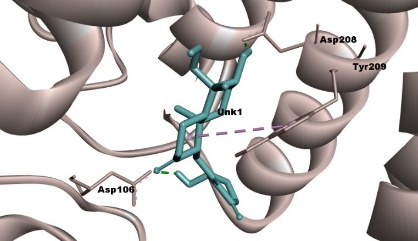


**B)**

**Fig. 8** Structural analysis for ligand-binding regions of AdipoR1 from sequential docking of gallic acid followed by andrographolide. (A) The position of gallic acid (purple) and andrographolide (cyan) on AdipoR1. (B) Detailed view of the molecular interactions of andrographolide with AdipoR1 residues.

**Supplementary 4:**


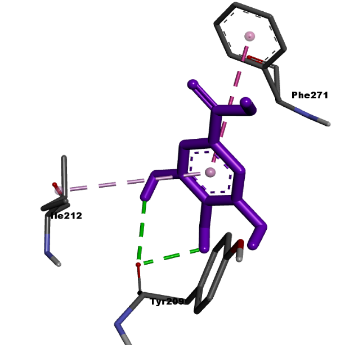

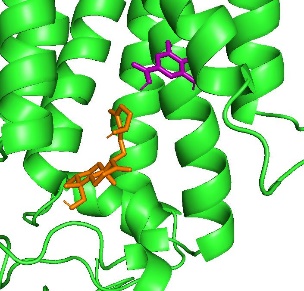


**A)**

**B)**

**Fig. 9** Structural analysis for ligand-binding regions of AdipoR1 from sequential docking of andrographolide followed by gallic acid. (A) The position of gallic acid (purple) and andrographolide (orange) on AdipoR1. (B) Detailed view of the molecular interactions of gallic acid with AdipoR1 residues.
